# Insect repellents mediate species-specific olfactory behaviours in mosquitoes

**DOI:** 10.1186/s12936-020-03206-8

**Published:** 2020-03-30

**Authors:** Ali Afify, Christopher J. Potter

**Affiliations:** The Solomon H. Snyder Department of Neuroscience, The Center for Sensory Biology, Johns Hopkins University School of Medicine, Baltimore, MD 21205 USA

**Keywords:** Spatial repellents, Human odorants, Olfaction, Behaviour, Calcium imaging, Q-system, Olfactory neurons, Masking

## Abstract

**Background:**

The species-specific mode of action for DEET and many other mosquito repellents is often unclear. Confusion may arise for many reasons. First, the response of a single mosquito species is often used to represent all mosquito species. Second, behavioural studies usually test the effect of repellents on mosquito attraction towards human odorants, rather than their direct repulsive effect on mosquitoes. Third, the mosquito sensory neuron responses towards repellents are often not directly examined.

**Methods:**

A close proximity response assay was used to test the direct repulsive effect of six mosquito repellents on *Anopheles coluzzii*, *Aedes aegypti* and *Culex quinquefasciatus* mosquitoes. Additionally, the behavioural assay and calcium imaging recordings of antennae were used to test the response of *An. coluzzii* mosquitoes towards two human odorants (1-octen-3-ol and benzaldehyde) at different concentrations, and mixtures of the repellents lemongrass oil and *p*-menthane-3,8-diol (PMD) with DEET.

**Results:**

*Anopheles coluzzii* mosquitoes were repelled by lemongrass oil and PMD, while *Ae. aegypti* and *Cx. quinquefasciatus* mosquitoes were repelled by lemongrass oil, PMD, eugenol, and DEET. In addition, high concentrations of 1-octen-3-ol and benzaldehyde were repellent, and activated more olfactory receptor neurons on the *An. coluzzii* antennae than lower concentrations. Finally, changes in olfactory responses to repellent mixtures reflected changes in repulsive behaviours.

**Conclusions:**

The findings described here suggest that different species of mosquitoes have different behavioural responses to repellents. The data further suggest that high-odour concentrations may recruit repellent-sensing neurons, or generally excite many olfactory neurons, yielding repellent behavioural responses. Finally, DEET can decrease the neuronal and behavioural response of *An. coluzzii* mosquitoes towards PMD but not towards lemongrass oil. Overall, these studies can help inform mosquito repellent choice by species, guide decisions on effective repellent blends, and could ultimately identify the olfactory neurons and receptors in mosquitoes that mediate repellency.

## Background

Female mosquitoes can carry a number of deadly infectious agents transmittable to humans via a bite. In 2017, approximately 700,000 deaths occurred as a result of mosquito bites from three divergent species of mosquitoes (*Anopheles*, *Aedes* and *Culex*) [1]. Mosquitoes use their sense of smell to seek out and distinguish a vertebrate host for a blood meal. Disturbing a mosquito’s sense of smell can reduce host-seeking behaviours. The use of insect repellents, which can alter olfactory responses in mosquitoes, is one strategy of personal protection from host-seeking mosquitoes. There are two broad categories of insect repellents available on the market: products containing synthetic repellents (DEET, IR3535 or picaridin) or products containing natural plant-based repellents (e.g., oil of lemon eucalyptus, eugenol, lemongrass oil). Since the 1950s, DEET has been the gold standard in mosquito repellents [2]. However, the mode of action for DEET, and most mosquito repellents, has been unclear. There are three hypotheses of how DEET affects a mosquito’s host-seeking behaviour: (1) DEET activates chemoreceptors on the mosquito antennae, maxillary palps, or the labella to repel mosquitoes (‘smell and avoid’) [3–9]; (2) DEET modulates chemoreceptor activity in response to attractive odorants (‘scrambling’) [10–12]; (3) DEET works directly on the odorants to decrease their volatility leading to a decreased amount of the odorants reaching the mosquitoes (‘masking’) [4, 13]. In addition to its role in affecting mosquito olfaction, DEET also functions as a robust contact repellent in *Aedes*, which requires the tarsus to trigger avoidance [14].

**Fig. 1:**
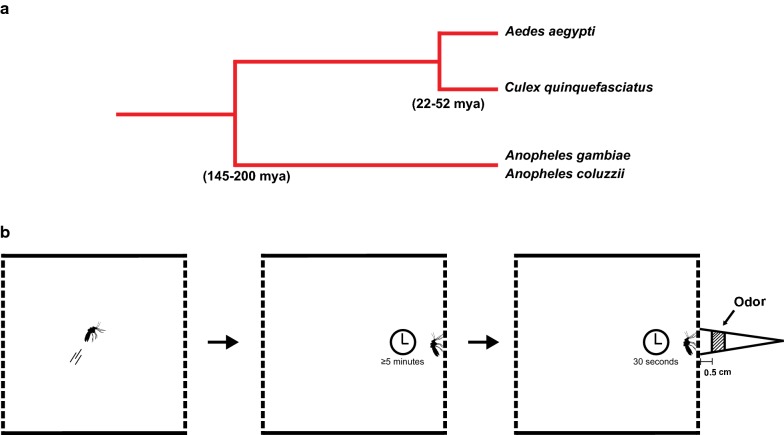
Close proximity response assay. **a** Phylogenetic relationship of the three mosquito species used in this study (adapted from Sieglaff et al. [17]). **b** A schematic of the close proximity response assay. An individual mosquito is introduced into the cage. The mosquito lands on the mesh wall of the cage and is allowed to rest for at least 5 min for acclimatization, before starting the experiment. A pipette tip containing a piece of filter paper soaked with the odorant is placed on the opposite side of the mesh wall (filter paper is 0.5 cm away from the mosquito), and the mosquito is monitored for 30 s

There are approximately 3500 species and sub-species of mosquitoes belonging to two sub-families, the Anophelinae (e.g. *Anopheles* mosquitoes) and Culicinae (e.g. *Culex* and *Aedes* mosquitoes) [15]. Anophelinae and Culicinae diverged between 145 and 200 million years ago [16, 17] (Fig. 1a), which is sufficient time for insect olfactory systems to evolve independently and respond differently to odorants, including repellents [18–25]. Therefore, the lack of agreement on how DEET and other mosquito repellents work from previous studies might reflect the assumption and use of a single mosquito species as representative for all mosquito species [4, 6–11, 13]. In addition, to behaviourally test the effect of repellents on host seeking, previous studies typically used human odours as attractants and measured the change in attraction in the presence of the repellent [3, 6, 8, 26–28]. In these assays, synthetic repellents interact with human odorants and decrease their volatility [4, 13], leading to an indirect inhibition of chemoreceptor responses towards human odorants (chemical masking). A simple olfactory behavioural assay was recently developed to monitor the response of *Anopheles* mosquitoes towards repellent odours alone [13]. In this close proximity response assay (Fig. 1b), DEET odours did not directly repel *Anopheles* mosquitoes [13]. This work raised several questions. Would DEET odours directly repel other mosquito species in this assay? Do other synthetic repellents, like IR3535 or picaridin, act the same way as DEET? How effective are natural plant-based repellents, and how might they affect various mosquito species differently? In this work, the olfactory responses of three mosquito species (*Anopheles coluzzii*, *Aedes aegypti* and *Culex quinquefasciatus*) were directly tested to synthetic (DEET, IR3535, picaridin) and natural repellents (lemongrass oil, eugenol, and the active ingredient in oil of lemon eucalyptus *p*-menthane-3,8-diol) in the close proximity response assay. The three mosquito species differed in their responses to synthetic and natural repellents. Furthermore, even human odorants, such as 1-octen-3-ol and benzaldehyde, could elicit repulsion at high concentrations. Using calcium imaging in transgenic *Anopheles* mosquitoes, higher concentrations of these odorants increased olfactory neuron responses and activated additional olfactory neurons (in comparison to low concentrations of odorants). In addition, in order to correlate olfactory neuron responses with olfactory-driven behaviours in the close proximity assay, transgenic *An. coluzzii* mosquitoes were utilized to directly assay the olfactory neuron responses to repellent mixtures and to determine whether visualized olfactory neuron responses of repellent mixtures might be predictive of behavioural responses [13]. Olfactory neuron imaging indicated a more dramatic decrease in the ability of PMD to activate olfactory neurons compared to lemongrass oil, and this was similarly reflected in repellent behavioural responses to the repellent mixtures.

## Methods

### Mosquitoes

*Anopheles coluzzii* (Wild type Ngousso strain; genotype: *Orco*-*QF2* [29]*, QUAS*-*GCaMP6f* [13]), *Aedes aegypti* (Wild type LVPib12 strain), and *Culex quinquefasciatus* mosquitoes (Wild type Johannesburg strain) were raised in a climate chamber maintained at 26–28 °C, 70–80% RH and L14:D10 cycle. Eggs were hatched in de-ionized water, and larvae fed on fish food (TetraMin^®^), added daily. Three days after hatching, larvae were counted and kept at a density of ~ 150 larvae/L of water. Cotton rolls soaked with a sugar solution (10% w/vol) were provided to feed adult mosquitoes. Colony female mosquitoes were blood fed on mice according to a protocol approved by the Johns Hopkins University Animal Care and Use Committee. For all experiments, non blood-fed female mosquitoes (3–10 days old) that were allowed to mate freely were used.

### Odorants

Odorants were purchased at the highest purity available. 1-octen-3-ol (SAFC, product # W280518), and benzaldehyde (Aldrich, product # 418099) were used undiluted or diluted in paraffin oil (Sigma-Aldrich, product# 18512) to 0.1, 1 and 10%. IR3535 (EMD Chemicals, product# 111887), picaridin (Cayman Chemical, product# 16458), and eugenol (Aldrich, product# E51791) were used undiluted. Lemongrass oil (SAFC, product# W262404), *p*-menthane-3,8-diol (BOC Sciences, 80%, catalog# B0005-092293), and DEET (Sigma Aldrich, product # 36542), were used undiluted, or diluted in paraffin oil to 30%.

### Behaviour

#### Close proximity response assay

Female mosquitoes were tested individually (a total of 30 mosquitoes for each experiment). A mosquito was transferred to a cage (BugDorm, 30 × 30 × 30 cm) and given enough time (≥ 5 min) to come to rest on one of the cage mesh walls (Fig. 1b). After 30 s at rest, the mosquito was then approached from outside the cage by a 1000 μl pipette tip (Denville) containing a piece of filter paper (1 × 2 cm) soaked with 20 µl of the test odorant. The pipette tip, held by a gloved hand, was rested on the outside of the cage wall so that the mosquito was at a 0.5 cm distance from the filter paper. The mosquito was observed for 30 s and the time at which it flew away was scored. The sequence of the odorants was randomized every time, and the mosquito was given ≥ 2 min between odorants. A mosquito that flew off in response to an odorant was allowed to land and rest for ≥ 2 min before the next odorant was used.

#### Analysis of close proximity response assay

A ‘Kaplan–Meier Survival Estimates’ was used to summarize the time that all 30 tested mosquitoes took to fly in response to odorants. A Cox Proportional Hazard Model was then used to assess significant differences in response time, which also considered the number of previous odorant exposures. The plot and analysis were performed using ‘survival’ and ‘survminer’ packages in R [30].

### Calcium imaging

#### Mosquito preparation

Only female mosquitoes were used. A mosquito was immobilized on ice for 1 min, inserted into a pipette tip, and pushed so that only its antennae extended outside the pipette tip. The pipette tip was then mounted onto a glass slide using modelling clay. The antenna was placed forward and held down on a glass cover slip using two pulled glass capillary tubes (Harvard Apparatus, 1 OD × 0.5 ID × 100 L mm). One tube was used to hold down the 3rd–4th antennal segment, and the other glass tube was used to hold down the 12th–13th segment (the most distal segments). All recordings focused on one antennal segment (11th antennal segment). Previous recordings found that the responses of this one segment (11th segment) were representative of olfactory responses from the other segments [13].

#### Imaging system

Imaging was performed through a 50× objective (LD EC Epiplan-Neofluar 50×/0.55 DIC) mounted on a Zeiss Axio Examiner D1 microscope. A Zeiss Illuminator HXP 200C light source and an eGFP filter cube (FL Filter Set 38 HE GFP shift free) were used for fluorescence.

An EMCCD camera (Andor iXon Ultra, Oxford Instruments) using Andor Solis software (Oxford Instruments) was used to record videos of 20 s at 512 × 512 pixel resolution. The exposure time was 200 ms (5 Hz).

#### Odorant preparation and delivery

For testing human-odorants, 20 µl of the odorant solution was pipetted onto a piece of filter paper (1 × 2 cm) that was placed in a Pasteur pipette (Fisher Scientific). For single repellents, 10 µl of the repellent (at 60%) was pipetted on the same filter paper with 10 µl paraffin oil. To prepare repellent mixtures, 10 µl of each repellent (at 60%) were pipetted on the same filter paper to reach the desired final concentration when mixed (30% of each repellent). The Pasteur pipette was inserted into a hole in a plastic serological pipette (Denville Scientific Inc, 10 ml pipette) carrying a continuous stream of purified air (8.3 ml/s) directed towards the antenna (Fig. 3b). A stimulus controller (Syntech) was used to divert a 1 s pulse of charcoal-filtered air (5 ml/s) into the Pasteur pipette 10 s after the beginning of each recording. The sequence of odorants was randomized for each set of experiments, and new Pasteur pipettes were prepared for each recording session.

#### Analysis of calcium imaging recordings

To generate heatmap ΔF images, Fiji software [31] was used with a custom-built macro. This macro uses the ‘Image stabilizer’ plug-in to correct for movement, followed by the ‘Z project’ function to calculate the mean baseline fluorescence. The mean baseline fluorescence was represented by the first 9 s of recording, just before stimulus delivery. The ‘Image calculator’ function was then used to subtract the mean baseline fluorescence from the maximum fluorescence frame after odorant delivery (this image was manually chosen). This calculated ΔF image was then used to produce the heatmaps in the Figures.

## Results

### Species-specific differences in mosquito behavioural response to repellents

**Fig. 2:**
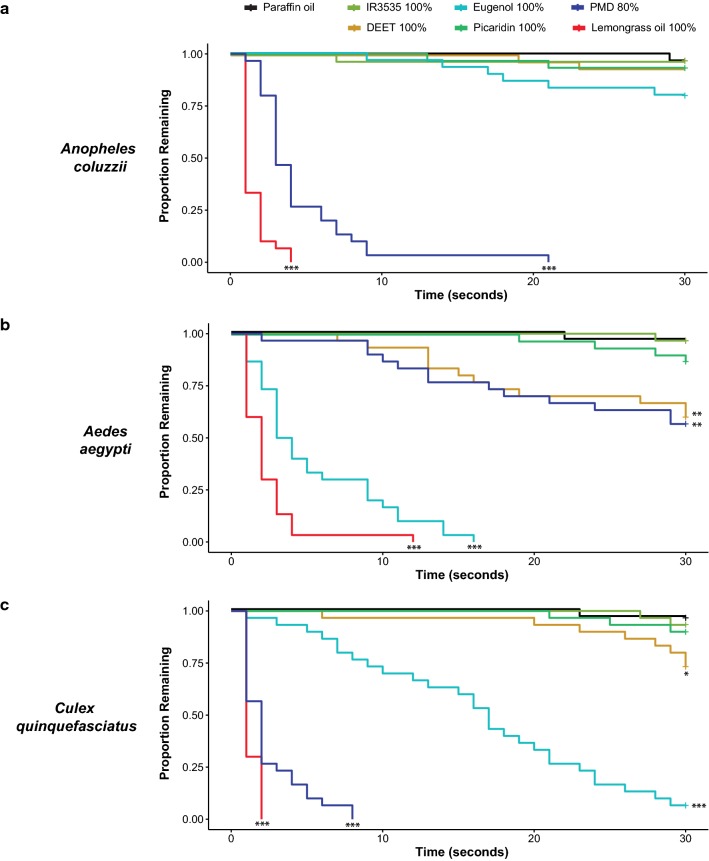
Species-specific behavioural responses to repellents. **a**–**c**, Kaplan–Meier estimates show the proportions of *Anopheles coluzzii* (**a**), *Aedes aegypti* (**b**), and *Culex quinquefasciatus* (**c**) mosquitoes that remained on the cage wall over time in response to repellents and the paraffin oil control (n = 30 mosquitoes for each species). Asterisks indicate significant differences from paraffin oil (Cox Proportional Hazard Model, *P < 0.05, **P < 0.01, ***P < 0.001)

DEET does not directly activate odorant receptors (ORs) in *An. coluzzii* mosquitoes, and does not directly repel them [13]. This is in contrast to what has been reported for *Ae. aegypti* [6–8] and *Cx. quinquefasciatus* [4, 9] mosquitoes. Therefore, the direct effect of DEET (not in contact with other odorants) was tested in the close proximity response assay on these two mosquito species (Fig. 2). In addition, the behavioural effect of other commonly used synthetic repellents (IR3535 and picaridin) as well as three natural repellents (lemongrass oil, eugenol, and PMD) were tested on all three mosquito species. Consistent with previous findings [4, 6, 8, 9, 13, 26, 28], DEET did not repel *An. coluzzii* mosquitoes, but was mildly repulsive to both *Ae. aegypti* and *Cx. quinquefasciatus* mosquitoes (Fig. 2). The synthetic repellents IR3535 and picaridin were not repulsive to any of the mosquito species tested. The greatest behavioural differences across the mosquito species were in their responses to natural repellents. *Anopheles coluzzii* mosquitoes were repelled by lemongrass oil and PMD only (Fig. 2a). Eugenol showed a weak repellent effect to *Anopheles* mosquitoes, but it was not significantly different than paraffin oil (P = 0.08, Fig. 2a). In contrast, *Ae. aegypti* and *Cx. quinquefasciatus* mosquitoes were repelled by lemongrass oil, PMD, and eugenol (Fig. 2b, c). PMD was more repellent to *Anopheles* and *Culex* mosquitoes than to *Aedes* mosquitoes, whereas eugenol was more repellent to *Aedes* mosquitoes than to *Anopheles* or *Culex* mosquitoes. Differences in repellencies might reflect species-specific differences in their olfactory receptor neurons to respond to these odours.

### Olfactory behavioural responses to human odorants measured by the close proximity assay

**Fig. 3:**
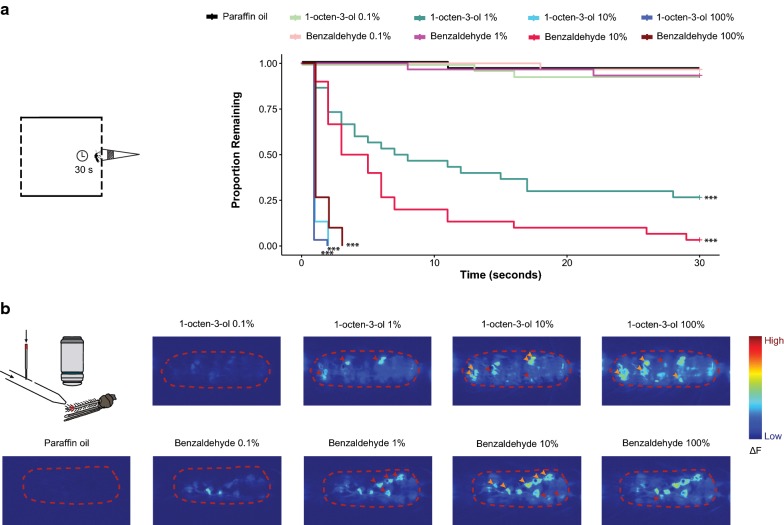
Response of *Anopheles coluzzii* mosquitoes to different concentrations of human odorants. **a** Kaplan–Meier estimate shows the proportion of mosquitoes that remained on the cage wall over time in response to test odorants and the paraffin oil control (n = 30 mosquitos). Asterisks indicate significant differences from paraffin oil (Cox Proportional Hazard Model, ***P < 0.001). **b** (left, top) A schematic of the calcium imaging setup. (left, bottom) Example heatmap from calcium imaging recording shows the response to paraffin oil (control). (right) Example heatmaps from calcium imaging recordings show the responses to 1-octen-3-ol and benzaldehyde at 0.1, 1, 10, and 100% concentrations. Red arrowheads point to neurons that start responding at higher odorant concentrations, and orange arrowheads point to neurons that showed stronger responses at higher concentrations

A better understanding of how odours repel mosquitoes could lead to the generation of improved spatial repellents. To reach this goal, it is necessary to identify concentrations at which an odorant becomes repellent, and understand how this switch happens at the neural level. Work in *Drosophila* has suggested that even attractive odorants can become repellent at high concentrations [32, 33]. For example, the strong attractant apple cider vinegar became less attractive at a higher concentration due to the additional activation of an olfactory receptor neuron at the high odour concentration [32]. This raises the question if human odorants (which are often attractants) might also become repellent to mosquitoes at high concentrations. To address this, the close proximity assay was used to test the behavioural response of *An. coluzzii* mosquitoes towards two human skin odorants, 1-octen-3-ol and benzaldehyde, at a range of concentrations (0.1, 1, 10, and 100%). At 0.1% concentrations for both odorants, *Anopheles* mosquitoes did not respond in this assay (Fig. 3a). In contrast, 1-octen-3-ol caused mosquitoes to fly away at 1, 10 and 100% concentrations, while benzaldehyde caused mosquitoes to fly away only at 10 and 100% concentrations (Fig. 3a). These data suggest that *Anopheles* mosquitoes may become behaviourally repelled to high host-odorant concentrations, suggesting that host-odour concentrations might need to be in narrow concentration ranges to attract mosquitoes. These data further suggest that changes in olfactory behaviours towards higher odour concentrations might be reflected by changes to olfactory neuron responses.

### Higher odorant concentrations recruit additional olfactory neurons

The concentration of 1-octen-3-ol in human sweat is typically 0.49 µg/ml [34]. Although it is difficult to directly compare odour stimulations, the repellent concentration of 1% 1-octen-3-ol is likely high in comparison with the concentration of 1-octen-3-ol found in human sweat [34]. To begin to address how high concentrations of non-repellent odorants might drive mosquito repulsion, calcium imaging was used to examine the *Anopheles* mosquito antennal response towards 1-octen-3-ol and benzaldehyde at 0.1, 1, 10, and 100% concentrations. Transgenic mosquitoes were used in which the calcium indicator GCaMP6f was expressed in all neurons that express the odorant receptor coreceptor Orco (genotype: *Orco*-*QF2*, *QUAS*-*GCaMP6f* [13]). This enabled direct monitoring of responses in olfactory neurons (Orco + neurons) towards the test odorants. Low concentration of both odorants elicited specific patterns of olfactory neuron activities, which presumably reflect olfactory neurons expressing odorant receptors most sensitive to these odorants. Higher concentrations of both odorants elicited stronger responses in the same antennal olfactory neurons (Fig. 3b, orange arrowheads). Interestingly, at higher odorant concentration, more neurons responded than at low concentrations (Fig. 3b, red arrowheads). This suggests that higher concentrations of non-repellent odorants not only more robustly activate highly sensitive olfactory neurons, but might recruit low-sensitivity, and potentially repellent-activated, neurons. It is also possible that widespread activation of many olfactory neurons might be interpreted by the mosquito olfactory system as a repellent signal [35]. Overall, these data highlight olfactory neuron activity patterns that might be linked to repellent responses.

### Repellent mixing modulates the odour potency of repellent components

**Fig. 4:**
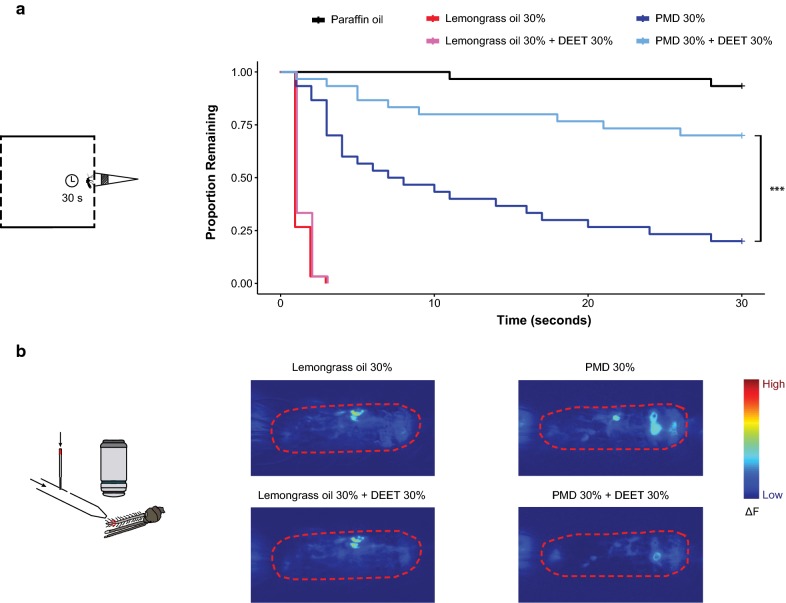
Responses towards mixtures of natural repellents and DEET. **a** Kaplan–Meier estimate show the proportions of *An. coluzzii* mosquitoes that remained on the cage wall over time in response to lemongrass oil, PMD, their mixtures with DEET, and the paraffin oil control (n = 30 mosquitoes). Asterisks indicate significant differences between a repellent and its mixture with DEET (Cox Proportional Hazard Model, ***P < 0.001). **b** Example heatmaps from calcium imaging recordings show the responses towards lemongrass oil, PMD, and their mixtures with DEET

By reducing the volatility of odorants with which they are mixed, synthetic repellents like DEET can function to ‘hide’ human odours from host-seeking mosquitoes [13]. However, this also suggests that spatial repellents, when mixed with DEET, might not be as effective at repelling mosquitoes than the spatial repellents alone. Prior studies found that DEET prevented eugenol from strongly activating olfactory neurons but did not affect the response to lemongrass oil in calcium imaging experiments [13], suggesting that lemongrass oil may still function as a spatial repellent in mixtures with DEET. To address this, the behavioural effect of mixing DEET with the natural repellents lemongrass oil and PMD was examined (Fig. 4). DEET did not change the response of *An. coluzzii* mosquitoes towards lemongrass oil; all *An. coluzzii* mosquitoes were similarly repelled by lemongrass oil (30%) and a mixture of lemongrass oil (30%) and DEET (30%) (Fig. 4a). On the other hand, adding DEET to PMD significantly decreased the mosquito repulsion mediated by PMD alone, although the response to the PMD + DEET mixture was still significantly different from paraffin oil (P = 0.04, Fig. 4a). Given that DEET can function to reduce odour signalling suggests that calcium imaging of olfactory neurons might experimentally allow behavioural changes to be correlated with olfactory neurons responses. To test this, calcium imaging of olfactory neurons was performed to test antennal responses to PMD, lemongrass, and their mixtures with DEET. Lemongrass oil and PMD strongly activated ~ 2–5 olfactory neurons in antennal segment 11. When mixed with DEET, lemongrass oil was still able to robustly activate the same olfactory neurons (Fig. 4b). On the other hand, when PMD was mixed with DEET, the olfactory responses to PMD were strongly decreased, with less olfactory neurons being strongly activated (Fig. 4b). These results confirm previous findings demonstrating DEET’s ability to mask human odorants and other repellents [13], and further suggest that decreased behavioural responses to repellent mixtures can be predicted by the decreased olfactory neuronal responses to those mixtures.

## Discussion

Despite being used by hundreds of millions of people worldwide, the mechanisms by which mosquito repellents deter mosquitoes remain unclear. Three major factors of this confusion are proposed. First, behaviour of one mosquito species is often generalized to other divergent species, with no experimental evidence for such similarity. While these insects may indeed share some homologous receptors activated by insect repellents, it is also likely that their chemosensory receptors have diverged over millions of years of evolution, such that one species may be able to detect a chemical that promotes repulsion, while the others do not. This is likely the case for DEET; while *Aedes* and *Culex* mosquitoes appear to express odorant receptors that respond to DEET, adult *Anopheles* mosquitoes do not. Second, the sensory mechanism of action should be considered when discussing an insect repellent. Mosquito repellents may function at a distance (spatial repellents likely targeting the olfactory system) or upon contact (likely activating gustatory or other sensory systems). Or insect repellents may not modulate the function of olfactory neurons directly, but instead prevent other odours from activating olfactory neurons by reducing odour volatility at the skin surface. Since most testing for the efficacy of insect repellents examines only the final step in host-seeking (the number of mosquito bites), these various modes of action are often not distinguished, which can cause confusion when trying to assign a single function to an insect repellent. For example, an insect repellent such as DEET may function only as a contact repellent in *Anopheles* mosquitoes, as both a spatial and contact repellent in *Aedes* and *Culex* mosquitoes, and also as a chemical that interacts with host-odors and reduces their ability in activating mosquito’s olfactory neurons. Third, due to technical challenges, the effect of mosquito repellents on chemosensory receptor neurons has often been tested in in vitro heterologous systems or with proxy insects (such as *Drosophila*), which may not necessarily represent the same conditions as in the native mosquito species. While these experimental methods are convenient and valuable systems for formulating hypotheses on insect repellent functions, due to the complex nature of olfactory neurons in sensilla and the divergence of chemosensory systems, exogenous systems cannot substitute for examining the physiological responses of neurons in the mosquito species in question. This study attempted to address these three major areas of confusion by (1) using a simple behavioural assay to test repellents in three species of mosquitoes; (2) examining only odour-based responses towards a simplified odour-source; and (3) directly examining the olfactory neuronal responses in *Anopheles* mosquitoes towards these odours.

In the close proximity assay, *Anopheles* mosquitoes were repelled by lemongrass oil and PMD only, while *Aedes* and *Culex* mosquitoes were repelled, to varying degrees, by lemongrass oil, PMD, eugenol and DEET. These data suggest that the direct response of mosquitoes to repellents is species-specific, with clearer differences between Anophelinae (*Anopheles* mosquitoes) and Culicinae (*Aedes* and *Culex* mosquitoes) than within Culicinae itself. This is consistent with the predicted relatedness between the chemosensory systems among the three species; Anophelinae and Culicinae diverged 145–200 million years ago, while *Ae. aegypti* and *Cx. quinquefasciatus* diverged 22–52 million years ago [17]. The findings in this study also agree with previous reports that DEET does not repel adult *Anopheles* mosquitoes on its own [13], while directly repelling *Aedes* [6–8] and *Culex* mosquitoes [4, 9].

Commercial repellents usually contain a mixture of different active repelling compounds. Interactions between different compounds in a mixture can result in synergism (when the response to a mixture is greater than the sum response to its components), additivity (when the response to a mixture is equal to the sum response to its components), or antagonism (when the response to a mixture is less than the sum response to its components) [2]. Previous studies suggested that DEET masks the olfactory responses to eugenol but not to lemongrass oil as monitored by calcium imaging recordings [13]. Here, it was further shown that DEET, when mixed with lemongrass oil, does not alter the behavioural response of *An. coluzzii* mosquitoes towards lemongrass oil. On the other hand, DEET decreased the behavioural and olfactory neuron responses to PMD. This suggests that DEET may have an antagonistic effect on some repellents (such as PMD), while not affecting other repellents (such as lemongrass oil). However, at this point the possibility that DEET was able to mask PMD due to the weaker repulsive effect of PMD (as compared to lemongrass oil) rather than a specific antagonistic effect against PMD cannot be ruled out; DEET might be able to mask weaker spatial repellents but not stronger spatial repellents. Nevertheless, DEET can be used in mixtures with some other spatial repellents because of its effect as a potent contact repellent [14], in addition to its direct olfactory repellent effect on *Aedes* and *Culex* mosquitoes.

The behavioural potency of a spatial repellent is likely reflected in the activity of neurons in the mosquito olfactory system. By directly monitoring olfactory neuronal activity patterns and correlating these to behavioural responses, studies can begin to understand how the *Anopheles* olfactory system might be guiding repulsive behaviours. At high concentrations, two tested human odorants become repellent, and this was mediated by the recruitment of low-affinity olfactory neurons, in addition to increases in the activity of high-affinity neurons. Future studies could determine if repellency to high-odour concentrations is due to the recruitment of ‘repellent’ neurons (those normally activated by a spatial repellent) or a general response to over-activation of the olfactory system. Similarly, calcium imaging of olfactory responses to spatial repellents highlights a subset of neurons that likely play a key role in mediating mosquito repulsion. Lemongrass oil, for example, was a robust spatial repellent and only activated ~ 2–5 neurons in an antennal segment. PMD was also a strong spatial repellent, which activated 2–5 neurons alone, and fewer neurons when mixed with DEET. These olfactory neurons, and the odorant receptors they express, might be targeted by other natural spatial repellents, and could further serve as a bioassay for identifying odors that would serve as new spatial repellents.

## Conclusions

Species-specific differences in mosquito responses towards repellents were reported. Repellents such as lemongrass oil and PMD were able to directly repel mosquitoes from all the three species tested. On the other hand, repellents like DEET and eugenol directly repelled *Aedes* and *Culex* mosquitoes but not *Anopheles* mosquitoes. Human odorant, such as 1-octen-3-ol and benzaldehyde, can be repulsive to *An. coluzzii* mosquitoes at high concentrations, possibly due to activating more olfactory neurons on the mosquito antennae than lower concentrations. Finally, mixing repellents can either have an antagonistic effect, or a potentially additive effect. These results are important in deciding which repellents can be used against each species of mosquitoes, and whether mixing repellents could alter their repulsive effect.

## Data Availability

The imaging files and datasets generated and/or analysed during the current study are available from the corresponding author on request. Requests for resources and reagents should also be directed to the corresponding author, Christopher J. Potter (cpotter@jhmi.edu). *Anopheles* mosquito strains used in this study are available upon request or from BEI Resources (https://www.beiresources.org/AnophelesProgram/Anopheles.aspx).

## Abbreviations

PMD: *p*-menthane-3,8-diol
Orco: Odorant receptor coreceptor
